# In this Issue: Volume 113, Issue 9, September 2022

**DOI:** 10.1111/cas.14960

**Published:** 2022-09-09

## Development of a novel lactate dehydrogenase A inhibitor with potent antitumor activity and immune activation

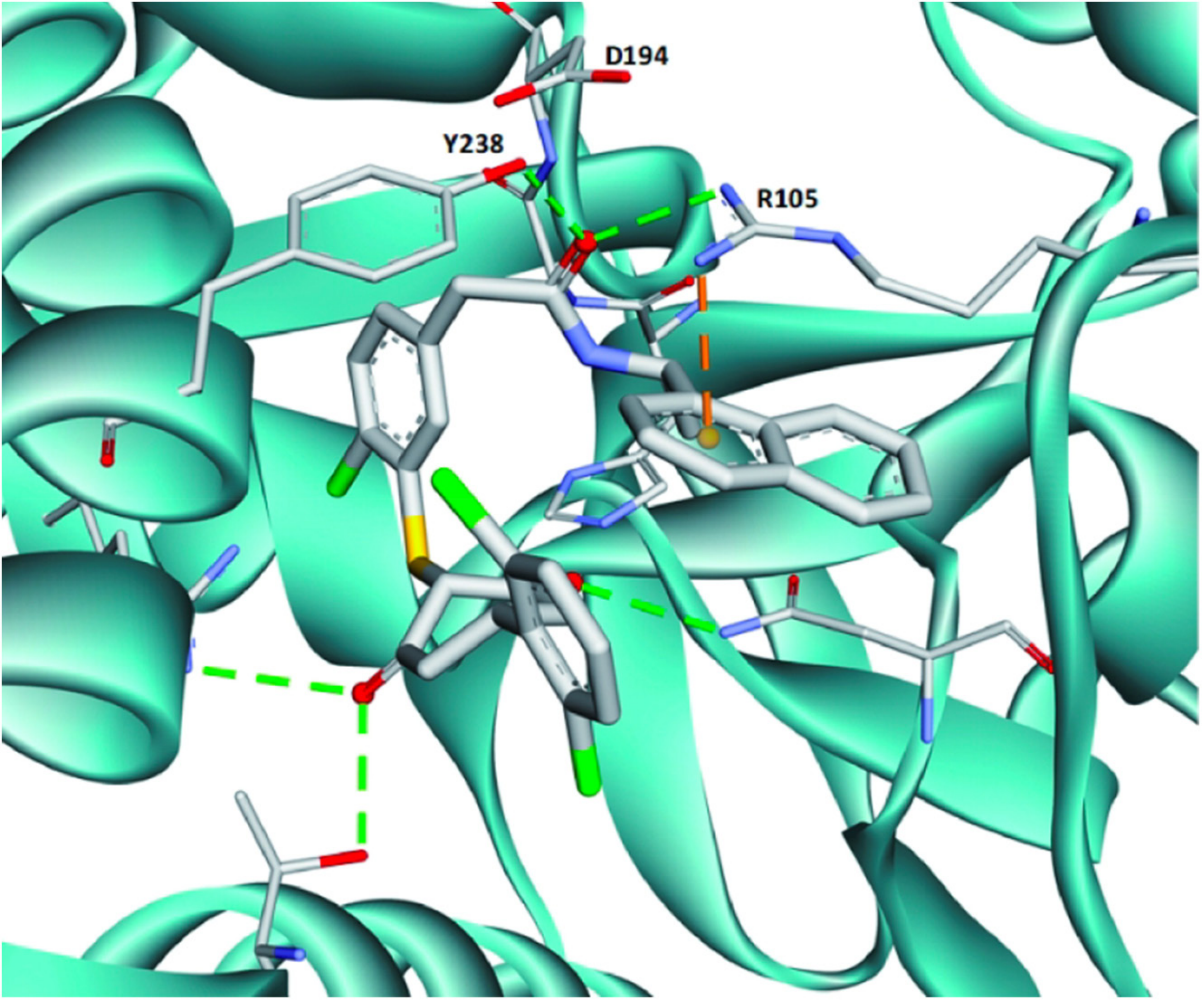

Cancer is a major cause of death around the world and affects millions of individuals globally. Unlike normal cells, cancer cells produce energy by converting sugars (i.e., glucose) to a compound called lactate. This process, famously known as the Warburg effect, leads to lactate accumulation in cancer cells. Subsequently, the elevations in lactate cause multiple adverse effects—including unchecked cell proliferation, increased blood vessel formation, and immune suppression—all of which promote cancer progression. Blocking lactate production, therefore, is a useful strategy in cancer therapy. One way to achieve this is the inhibition of lactate dehydrogenase A (LDHA), an enzyme that aids in lactate production. However, although various LDHA inhibitors have been developed, their efficacy against animal tumors has so far been limited.

Using a series of methods, ranging from cellular assays (in vitro) to animal model (in vivo) experiments, Du et al. designed a novel LDHA inhibitor, ML‐05, and tested its efficacy in their study. Their findings revealed that ML‐05 could inhibit lactate production in cultured cells quite effectively, thereby reducing the toxic effects of lactate accumulation. Further, the researchers showed that their LDHA inhibitor was capable of arresting cell division at the G1 phase, a crucial checkpoint in the cell cycle, thus preventing uncontrolled cell division.

In addition to the cellular effects, ML‐05 also showed strong antitumor effects in mouse models. Interestingly, this effect could be achieved by the direct local injection of ML‐05 at the tumor site, without the need for oral intake or systemic administration. ML‐05 also increased the population and activity of two specific kinds of immune cells, namely, Th1 and GMZB^+^CD8 T cells, which further amplified its antitumor effects. Moreover, ML‐05 also enhanced the antitumor effects of the anti‐programmed cell death‐1 (PD‐1) antibody, an important immunotherapy agent used in cancer treatment.

Taken together, these results show that the novel LDHA inhibitor, ML‐05, is an immensely potent antitumor agent, both in vitro and in vivo. Additionally, the study suggests that ML‐05 can enhance the effects of immunotherapy, and consequently, it carries a significant translational value.

https://onlinelibrary.wiley.com/doi/full/10.1111/cas.15468

## TIFA promotes colorectal cancer cell proliferation in an RSK‐and PRAS40‐dependent manner

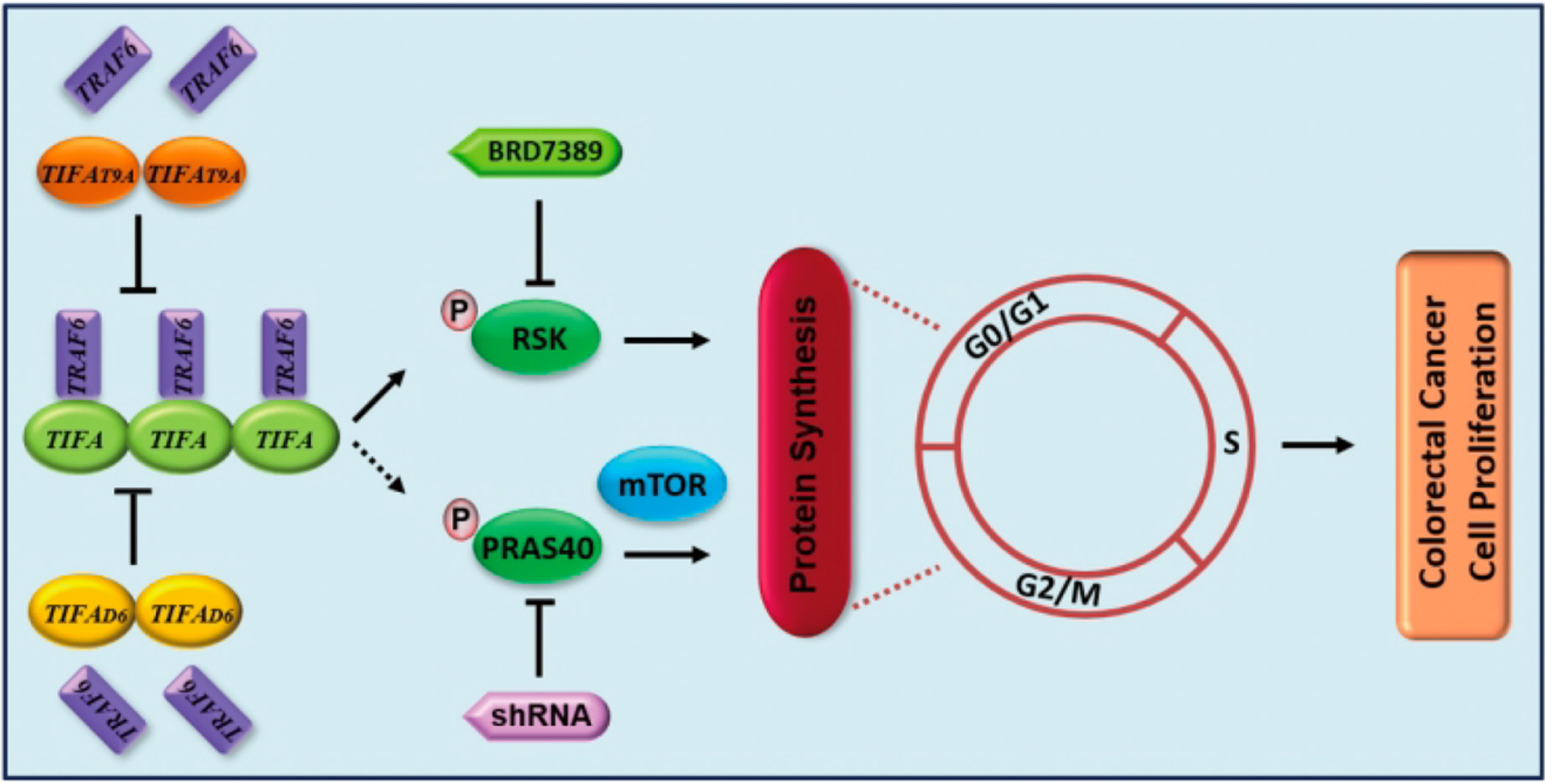

Colorectal cancer (CRC), caused by the uncontrolled proliferation of cells in the colon or rectum, is one the most common cancers with over 1.4 million new cases reported every year. Advances in screening and treatment have improved the survival rates of patients with CRC. However, treatment strategies targeting tumor progression, metastasis (spread to distant organs) and recurrence are lacking, thus underscoring the need to identify novel cellular targets for CRC treatment.

Previous studies have demonstrated the role of a protein named “TIFA”, in promoting cancer cell ‐ migration, proliferation and survival, in lung and liver cancer. However, its involvement in CRC remains unexplored. In this study, Shen et al. explored the potential roles of TIFA in CRC pathogenesis using patient derived tumor tissues, CRC cell lines and xenograft models (animals injected with human tumor cells).

They found that both tumor tissues and CRC cell lines had a higher expression level of TIFA compared to normal cells. Notably, the increase in TIFA expression correlated with the stage of the cancer, which reflects the degree of tumor invasiveness and metastasis.

Furthermore, cells deficient in TIFA exhibited decreased proliferation. Additionally, in mice injected with TIFA deficient cells, the authors noted a marked decrease in tumor growth and volume compared to controls. Conversely, “ectopic” (in addition to normal) expression of TIFA enhanced the proliferation of cultured cells and cells injected in mice.

Probing further, they found that an “oligomerization mutant” of TIFA (protein deficient in the sites required for linking of tandem units) failed to enhance cell proliferation, suggesting the mechanism underlying the role of TIFA in cell proliferation. The authors further propose that TIFA oligomerization is crucial for the activation of the proteins RSK kinase and PRAS40a, which mediate downstream signaling pathways associated with protein synthesis, a cardinal process for cancer cell proliferation and tumor progression.

Overall, these findings shed light on the role of TIFA and the mechanisms by which it contributes to CRC progression. The authors suggest that TIFA may serve as a novel therapeutic target for CRC treatment, as well as for predicting the course of the disease.

https://onlinelibrary.wiley.com/doi/full/10.1111/cas.15432

## *KRAS* variant allele frequency, but not mutation positivity, associates with survival of patients with pancreatic cancer

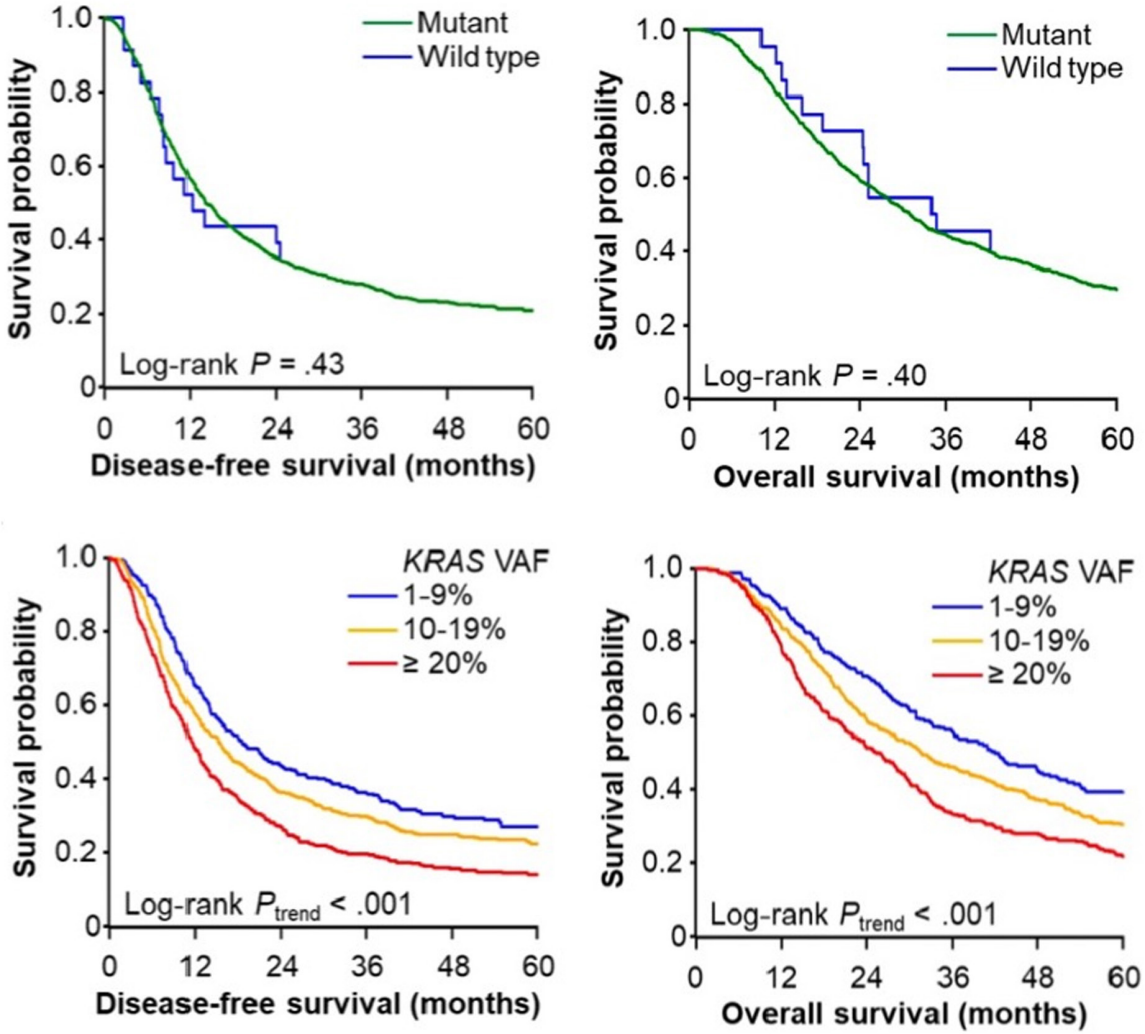

The *KRAS* gene encodes for the K‐Ras protein, which is involved in signaling pathways that govern the growth, maturation, and death of a human cell. Mutations in the *KRAS* gene can disrupt cell signaling, resulting in pancreatic cancer. Therapeutically targeting this mutated gene may help reverse cancer progression. However, the association between *KRAS* and patient survival has not been adequately investigated in large populations due to the lack of sensitive assays and sampling limitations.

Recently, the focus has shifted to droplet digital PCR (ddPCR), an emerging low‐cost technique that enables the sensitive detection and quantification of genetic mutations in tissues despite low sample amounts. This has enabled the detection of *KRAS* variant allele frequency (VAF), which is a multifactorial index reflective of tumor count and associated mutation load, with higher accuracy.

To examine the feasibility of ddPCR in determining *KRAS* mutations and its associated survivorship, Suzuki et al. performed a multi‐institutional cohort study in a large pancreatic cancer patient population. Of the 1162 patients studied, 98% (1139) tested positive for the mutation. Notably, the authors achieved a comparable detection rate and identified *KRAS* mutations in tumor samples stored for up to 15 years! The findings successfully demonstrated ddPCR's sensitivity and detection efficiency regardless of tissue age, tumor cell count, or clinical variables.

The authors also established an inverse correlation between *KRAS* VAF and post‐operation survival periods in patients with resected pancreatic cancer using ddPCR quantification of variant alleles. They noted that this result remained consistent with the research that attributed cancer progression to increased *KRAS* mutations.

The authors suggest that tumors with high *KRAS* VAF levels are more likely to present the adenosquamous phenotype conferring poor survival outcomes. Interestingly, in contrast to earlier reports, they found no link between *KRAS* positivity and patient survival. It is speculated that *KRAS* VAF‐low tumors could have been misclassified as *KRAS* wild type (non‐mutated) tumors due to the lack of sensitive assays. This likely resulted in the overstating of patient survival times of *KRAS* mutated tumors and therefore warrants validation using sensitive techniques like ddPCR. Therefore, *KRAS* VAF levels, rather than *KRAS* mutation positivity, serve as a more accurate predictor of patient survival in pancreatic cancer.

Overall, the study confirmed the prognostic value of *KRAS* mutation load and the utility of ddPCR as a first‐line analytical method for genomic profiling in pancreatic cancer patients, particularly for individualized care. https://onlinelibrary.wiley.com/doi/full/10.1111/cas.15398

